# Effect of Oxalic Acid Concentration and Different Mechanical Pre-Treatments on the Production of Cellulose Micro/Nanofibers

**DOI:** 10.3390/nano12172908

**Published:** 2022-08-24

**Authors:** Gabriela Adriana Bastida, Carla Natalí Schnell, Paulina Mocchiutti, Yamil Nahún Solier, María Cristina Inalbon, Miguel Ángel Zanuttini, María Verónica Galván

**Affiliations:** Institute of Cellulosic Technology, Faculty of Chemical Engineering (FIQ-CONICET), National University of the Litoral, Santiago del Estero 2654, Santa Fe S3000AOJ, Argentina

**Keywords:** oxalic acid, refining, rotary homogenizer, intrinsic viscosity, aspect ratio, gel point

## Abstract

The present work analyzes the effect of process variables and the method of characterization of cellulose micro/nanofibers (CMNFs) obtained by different treatments. A chemical pre-treatment was performed using oxalic acid at 25 wt.% and 50 wt.%. Moreover, for mechanical pre-treatments, a rotary homogenizer or a PFI mill refiner were considered. For the mechanical fibrillation to obtain CMNFs, 5 and 15 passes through a pressurized homogenization were considered. The best results of nanofibrillation yield (76.5%), transmittance (72.1%) and surface charges (71.0 µeq/g CMNF) were obtained using the PFI mill refiner, 50 wt.% oxalic acid and 15 passes. Nevertheless, the highest aspect ratio (length/diameter) determined by Transmission Electron Microscopy (TEM) was found using the PFI mill refiner and 25 wt.% oxalic acid treatment. The aspect ratio was related to the gel point and intrinsic viscosity of CMNF suspensions. The values estimated for gel point agree with those determined by TEM. Moreover, a strong relationship between the intrinsic viscosity [η] of the CMNF dispersions and the corresponding aspect ratio (p) was found (ρ[η] = 0.014 p^2.3^, R^2^ = 0.99). Finally, the tensile strength of films obtained from CMNF suspensions was more influenced by the nanofibrillation yield than their aspect ratio.

## 1. Introduction

The depletion of petroleum products and the need to reduce the carbon footprint promote the development of new biodegradable materials obtained from renewable sources. Cellulose is the most abundant natural polymer that can provide rational solutions to these problems. In the last decades, the production of nanocellulose obtained from the cell walls of wood fibers and plants has gained great interest due to its interesting properties, such as high tensile strength and stiffness, low weight, renewability, biocompatibility, biodegradability and non-toxicity [1,2,3]. Consequently, cellulose nanofiber (CNF) can be used in several applications, such as reinforcement in biocomposites [4], biomedical application [5], strength additive in papermaking [6] and aerogels [7].

CNFs are usually prepared by applying an intense mechanical treatment, in which the fibers are subjected to great shearing forces that fibrillate and release microfibrils. The most common mechanical treatments include repeated steps in high-pressure homogenizers [8], microfluidizers [9], colloid mills [10], twin-screw extrusion [11] and high-intensity ultrasonication [12].

Nevertheless, a mechanical, chemical or enzymatic pre-treatment of fibers can also be applied to partially separate them and prevent clogging, as well as to reduce the energy consumption in the homogenizer [3,13]. 

Among mechanical pre-treatments, PFI refiners were found to produce high internal fibrillation, facilitating the subsequent mechanical treatment [8]. Ultra-turrax homogenization can also be used as a mechanical pre-treatment to improve nanofibrillation yields and their dispersibility [14,15].

2,2,6,6-Tetramethylpiperidine-1-oxyl (TEMPO)-mediated oxidation has been widely used as a chemical pre-treatment to obtain highly fibrillated CNFs [16,17,18]. However, this reagent is very expensive. Sanchez-Salvador et al. [19] reported that TEMPO and NaBr can be reduced by 67%, without altering the properties of the CNFs by reusing these reagents in the oxidation of cotton and eucalyptus cellulose. However, the cost of the reagent is still high and their disposal causes environmental concern.

Therefore, it is worth analyzing the application of other chemical pre-treatments different from TEMPO. One alternative is the use of organic dicarboxylic acids, such as oxalic acid. The treatment with this reagent produces carboxylation through Fischer–Speier esterification between one carboxylic group of the oxalic acid and one hydroxyl group on the accessible surface of the cellulose [20,21]. Oxalic acid has some advantages: it is cheap, it can be recovered and corrosion in equipment is controllable [3]. Chen et al. [21] obtained CNFs using oxalic acid at different concentrations, temperatures and reaction times. CNFs showed high thermal stability and a high aspect ratio, suggesting that they could be used as reinforcing component in biocomposites for different applications.

Normally, chemical pre-treatments different from TEMPO or the absence of chemical pretreatment lead to two fractions: CNF and microcellulose (CMF) one. Depending on the application considered, one of these fractions can be more useful than the other. For instance, Hu et al. [6] considered CNFs obtained by different mechanical method to analyze its performance as reinforcement of a recycled fiber paper. They found that CNFs with low nanofibrillation yield can produce a clear favorable effect provided that the aspect ratio (length/diameter) was high enough. The length of CNFs determines the probability that fibrils can be entrapped in the fiber network and can contribute to the increase in interfiber bonding.

Atomic Force Microscopy (AFM), Scanning Electron Microscopy (SEM), and Transmission Electron Microscopy (TEM) can be used to determine the diameter of the CNFs, but the length and, therefore, the aspect ratio is more difficult to evaluate due to entanglements of nanofibers [22]. There are different methods to estimate the aspect ratio of CNFs. Varanasi et al. [23] proposed the sedimentation method, which is based on the gel-point concentration. The gel point is the threshold consistency at which a continuous network of fibers in suspensions is formed. This method is an adaptation for nanocellulose of the method proposed by Martinez et al. [24] for wood pulp fibers. Another method involves the measurement of the intrinsic viscosity of cellulose micro/nano fiber (CMNF) suspensions. Albornoz-Palma et al. [25] found a relationship between this rheological parameter and the aspect ratio of enzymatic CNFs in a diluted regime. The use of simple and swift methods such as gel point and shear viscosity methodologies can be very useful to determine aspect ratios of these CMNFs based on dicarboxylic acid pre-treatments.

Our hypothesis is that variants in preparation conditions can have an impact in the nanofibrillation yield, charge density and aspect ratio, which are important parameters that define their potential applications. 

The purpose of the present study was to evaluate different pre-treatments for the production of oxalic-acid-based CMNFs. For this, the application of oxalic acid at two concentrations (25 wt.% and 50 wt.%) and two mechanical pre-treatments (a rotary homogenizer type ultra-turrax and a PFI mill refiner) were considered. After these pre-treatments, the fibers were fibrillated through the pressurized homogenizer considering 5 and 15 passes. Characteristics of CMNF suspensions like nanofibrillation yield, transmittance and surface charge were analyzed. The microfibrillar and nanofibrillar fractions were characterized by measuring the dimensions by SEM and TEM, respectively. The aspect ratio of the nanofibrillar fraction was related to two parameters of the CMNF suspension: gel point by sedimentation and intrinsic viscosity. Finally, the tensile strength of films obtained from CMNF suspensions was analyzed.

## 2. Materials and Methods

### 2.1. CMNF Preparation

CMNFs were obtained from industrial bleached eucalyptus pulp (BEP) supplied by Suzano Papel e Celulose S.A. (Aracruz, Brazil). The pulp was soaked in water for 24 h and disintegrated for 5 min at 1.5% consistency. The pulp suspension was centrifuged and stored at low temperature (4 °C) until use. The average dimensions of the hardwood fibers determined by optical microscopy were 15.6 µm in diameter and 0.95 mm in length. 

**Scheme 1:**
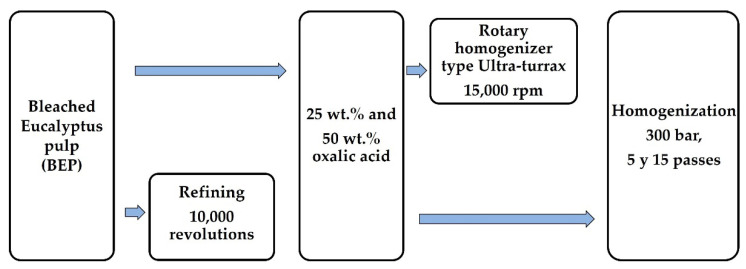
Stages of CMNF preparations.

Scheme 1 shows the stages of different cellulose micro/nanofibers obtained.

#### 2.1.1. Chemical Pre-Treatment

The chemical pre-treatment was carried out according to the technique described by Chen et al. [21]. In brief: 15 g of pulp were added to a reactor containing 750 mL oxalic acid considering two concentrations: 25 wt.% and 50 wt.%. The reaction was made at 90 °C for 1 h under constant stirring at 250 rpm. After that, the solution was filtered and the pulp was washed, starting with hot water to avoid the crystallization of the acid, and then, with water at room temperature until the conductivity of the filtrate was 20 µS/cm. Finally, the pulp was neutralized to pH 7.0 with NaOH solution, in order to obtain the sodium form of the carboxylic groups of the pulp.

#### 2.1.2. Mechanical Pre-Treatment 

Fibers chemically treated with oxalic acid were treated with a rotary homogenizer type ultra-turrax (Unidrive × 1000, Baden-Württemberg, Germany) at 0.75% consistency in distilled water, for 3 min/g fiber at 15,000 rpm.

On the other hand, BEP fibers were mechanically pre-treated using a Papirindustriens Forskningsinstitutt (PFI) mill at 10,000 revolutions and 10% of consistency in accordance with SCAN-C 18:65 standards. Then, the pulp was chemically pre-treatmented (as explained in Section 2.1.1).

#### 2.1.3. Mechanical Fibrillation

In all cases, the pulp was homogenized at 0.75 wt.% using a pilot scale pressurized homogenizer (SIMES S.A, Santa Fe, Argentina) at 300 bar, using 5 and 15 passes. The CMNFs obtained were stored at low temperature (4 °C) until use. 

Table 1 shows the identifications of the different cellulose micro/nanofibers prepared.

### 2.2. Characterization of CMNFs

Before making all characterization techniques, the CMNF solutions were sonicated for 2 min using a Sonics & Materials ultrasonic homogenizer (500 W, 40% amplitude) to ensure their adequate dispersion.

#### 2.2.1. Esterification and Carboxylation 

Cellulose carboxylation by the oxalic acid treatment was identified by Fourier Transform Infrared (FTIR) spectroscopy system (Shimadzu FTIR-8000 Spectrometer, (Shimadzu Co., Kyoto, Japan)). Samples of original fibers and the CMNF films were triturated and mixed with potassium bromide (KBr). Then, they were pressed into tablets. The spectra were recorded at the wavelength range of 400–4000 1/cm with a resolution of 8 1/cm. Each sample was scanned 40 times.

The carboxylic group content of the bleached eucalyptus pulp (BEP) and the CMNFs obtained with 25 wt.% and 50 wt.% oxalic acid treatment were determined by conductometric titration. For the original pulp, the technique proposed by Katz et al. [26] was used. For CMNFs, the determinations were made in a similar way to the method described by Ovalle-Serrano et al. [27]: a suspension was prepared by mixing 50 mg of CMNF in 400 mL of water. The pH was adjusted to 3.0 by adding 0.01M HCl solution to ensure a complete protonation of carboxyl groups. Conductometric titrations were performed using 0.01M NaOH solution as titrant under a nitrogen atmosphere.

#### 2.2.2. Nanofibrillation Yield, Transmittance and Surface Charges 

The yield of nanofibrillation was determined by centrifuging (4000 rpm, 2809 g) an aqueous suspension of 0.14 wt.% CMNF for 20 min according to Schnell et al. [28]. The dry weight of the supernatant, CNF, was obtained from the difference between the initial weight (*W_i_*) and the centrifugation sediment (*W_f_*) which was considered as CMF according to Equation (1):

(1) $$Yield\;\left(\%\right)=\left[\frac{W_{i}-W_{f}}{W_{i}}\right]*100$$

Transmittance readings of 0.1 wt.% CMNFs suspensions were performed at 800 nm using CECIL 3055 spectrophotometer and distilled water as reference. 

Surface charges of CMNFs were determined by polyelectrolyte titrations using streaming current measurements (Chemtrac ECA equipment, Norcross, GA, USA). A solution of 0.3 meq/L of cationic polyelectrolyte, poly(diallyldimethylammonium chloride) (M_w_ 400,000–500,000 Da) from Sigma Aldrich was used as titrant. The pH of CMNF suspensions (0.04 wt.% dissolved in 1 mM NaCl solution) was previously adjusted to 7.0.

#### 2.2.3. Morphologies

##### Measurements of Diameter and Length Distributions by Microscopy

The diameter and length distributions of CMNF fraction of diameter greater than 1 µm were obtained by optical microscopy (Leica Microsystems Instrument). Samples were diluted to 0.1 wt.%, and a single drop of the suspension was cast on a glass support and dried at room temperature. Then, 500 diameter and 50 length measurements were made using, for both, 40× and 100× lens.

The diameter distributions of CMF were examined using a Phenom Pro scanning electron microscope (SEM) (Eindhoven, Netherlands) at accelerating voltages of 5 KV and 10 KV, with a working distance of 2.5 ± 0.5 mm. The nanofibrillated fraction was removed by centrifugation, and the sediment was used for preparing suspensions of approx. 0.015 wt.%. The suspension was sonicated, lyophilized and the swollen CNF film formed was coated with gold. More than 75 diameter measurements were made using Image J processing software. The length measurements were not possible due to high entanglement of fibers.

The diameter and length distributions of CNF were determined by transmission electron microscopy (TEM) (JEOL, JEM-2100 Plus, Tokyo, Japan). Observations were made in HRTEM mode, with an acceleration voltage of 100 kV. The nanofibrillated fraction was removed by centrifugation as indicated above in Section 2.2.2. An aliquot of 0.001 wt.% of CNF suspensions was mounted on a carbon coated Cu grid. A number of 300 diameters and 75 length measurements were performed using Image J processing software. 

##### Sedimentation

According to the method proposed by Varanasi et al. [23], different concentrations of CMNFs were prepared (from 0.01 wt.% to 0.07 wt.%), the suspensions were stirred for 5 min and poured into graduated cylinders to reach an original suspension height (h_0_). After 48 h, the height of sediment in the cylinder (h_s_) was measured. The initial solid concentrations of CMNFs (C_0_) were plotted versus (h_s_/h_0_) and the data were adjusted to a quadratic equation. Then, the first derivate of the curve at the y-intercept gives the gel point (*C_c_*). For this method, the density of the cellulose nanofibers was assumed 1500 kg/m^3^. Then, the aspect ratio (*A*) can be estimated using Crowding Number theory (CN) with the following equation:

(2) $$A=6\;C_{c}{}^{-0.5}$$

This theory considers that the CNF are shaped like straight cylinders.

##### Shear Viscosity

The dynamic viscosity of different CMNF suspensions was determined using a Brookfield viscometer LVT with an Ultra Low Adapter (ULA) spindle similar to the method proposed by Albornoz-Palma et al. [25]. In brief, viscosity measurements were made at different concentrations (from 0.01 wt.% to 0.1 wt.%) and shear rate of 73.38 1/s. In all cases, samples were conditioned in a thermostatic bath at 25 °C for 1 h, and then, they were shaken for 1 min in a vortex before the measurement. 

The critical concentration (C*) was obtained from the changes in specific viscosity as a function of the CMNF concentration according to Tanaka et al. [29]. 

The intrinsic viscosity [η] was obtained in the dilute region extrapolating the linear fit to a zero concentration.

### 2.3. Film Formation from CMNFs

The formation of films was performed by adding 14 g of CMNF suspension at 0.75 wt.% in a petri dish. The samples were dried at 30 °C for 24 h. The thickness of the films was measured using a micrometer with a precision of 0.001. The average of 10 measurements of different parts of the film was reported. The mechanical properties of the CMNF films were measured using INSTROM 3340 equipment with a 100 N cell. First, the film was conditioned at 23 °C and 50%RH and then, 5 mm wide specimens were cut. For the mechanical tests, the ASTM D882 standard was followed. The initial distance between the grips was 22 mm and the tensile speed was 2 mm/min.

## 3. Results

### 3.1. CMNF Characterization

#### 3.1.1. Esterification and Carboxylation

**Figure 1:**
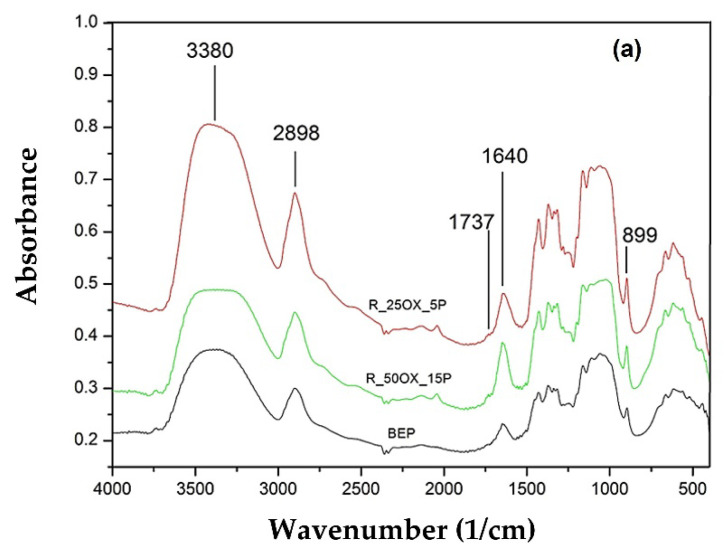

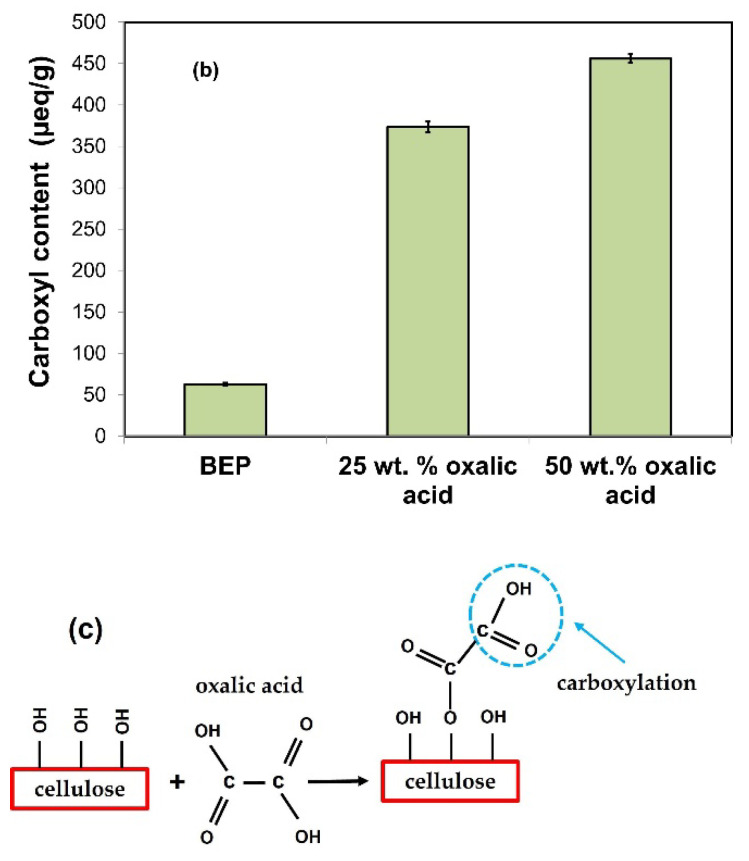
(**a**) FTIR spectra and (**b**) carboxylic content of the original bleached eucalyptus pulp (BEP) and the CMNF obtained from pulp treated with 25 wt.% and 50 wt.% oxalic acid. (**c**) Schematic diagram of cellulose carboxylation.

Figure 1a shows the infrared spectra of original BEP fibers and CMNFs pretreated with a PFI refiner and 25 wt.% and 50 wt.% oxalic acid. The spectra of the samples show the same cellulose characteristic peaks in 3380 1/cm and 2898 1/cm regions, attributed to the stretching vibration of hydroxyl groups (O-H) and the symmetric C–H stretching vibration, respectively [30,31]. The peaks around 1640 1/cm can be attributed to O–H bending vibration of the absorbed water and the peak at 899 1/cm can be assigned to β-glycosidic linkages of glucose ring in cellulose [32,33]. For the samples of CMNFs obtained by 25 wt.% and 50 wt.% oxalic acid treatment, a new weak peak was observed at 1737 1/cm, corresponding to the C=O ester vibrational stretching band [33]. This can be attributed to the reaction between the hydroxyl group on the cellulose chains and the carboxyl group of oxalic acid through Fischer–Speier esterification of one carboxyl group of oxalic acid (Figure 1c). 

On the other hand, Figure 1b shows the carboxylic group content obtained by conductometric titration of the bleached eucalyptus pulp (BEP) and the CMNFs pre-treated with PFI refiner and 25 wt.% and 50 wt.% oxalic acid. The total charge of CMNFs is expected to remain unaltered by mechanical treatments. Junka et al. [34] showed that the total charge of CNFs is independent of the number of passes through a microfluidizer. The results show that there is an important increase of the carboxyl group content for both CMNFs, also confirming the carboxylation of cellulose.

#### 3.1.2. Nanofibrillation Yield, Transmittance and Surface Charge 

Table 2 shows the nanofibrillation yield, the transmittance and surface charges of different CMNFs. The CNMFs corresponding to PFI refining pre-treatment had higher nanofibrillation yields, transmittance and surface charges than those obtained with a rotary homogenizer. Importantly, the refining step was carried out before the chemical pre-treatment because after oxalic acid treatment, the obtained paste cannot withstand a PFI refining. The mechanical pre-treatment prior to the chemical one, could have improved the accessibility between the hydroxyl groups of the cellulose and the carboxylic groups of the oxalic acid, thus increasing the nanofibrillation yield, transmittance and surface charges of the CMNF obtained. Moreover, the equipment used in the mechanical pretreatment also influences the characteristics of the CMNFs obtained. In the rotary homogenizer, a high-speed rotor forces the fiber suspension through the slots in the stator assembly. Minimal gap between rotor and stator produces extremely high shear forces that produces homogenization by dispersion. Additionally, these shear forces can cause fibrillation and fiber shortening. On the other hand, PFI mill refiner works by applying high shear forces to the suspension of fibers that are pressed between two parallel surfaces that rotate under constant load in the same direction. These causes internal and external fibrillation, generation of fines, and fiber shortening [35]. For CNMF preparation, internal fibrillation facilitates the final mechanical fibrillation process at the homogenizer. According to the results, while that the rotary homogenizer produces a greater number of fiber cuts, the PFI mill a greater internal and external fibrillation for these working parameters.

In addition, a significant increase in nanofibrillation yield was observed when the concentration of oxalic acid was increased from 25 wt.% to 50 wt.%. This result can be attributed to the new carboxylic groups introduced during the chemical treatment, which produce repulsive forces that facilitate the defibrillation of cellulose during the mechanical process [36]. Moreover, when the number of passes of the pressurized homogenizer was increased (from 5 to 15), no significant change in nanofibrillation yield was observed, except for the pulp treated with PFI and 50 wt.% oxalic acid (from 54.3% to 76.5%). This behavior could be associated with the working relatively low pressure (300 bar) of the homogenizer compared to those normally used (900–1000 bar), which hindered the increment in the main fibrillation.

On the other hand, Table 2 also shows that the transmittance values of the CMNF suspensions were similar and low for pulps treated with the rotary homogenizer (from 8.4% to 14.5%). However, for refined CNMFs, transmittance increased when the number of passes of the homogenizer and the concentration of oxalic acid were increased (from 18.7% to 72.1%). This increment is in agreement with a reduction in the residual fiber content and an increase in the content of optically inactive nanofibrils (higher nanofibrillation yield) in the CMNF suspensions. In addition, higher surface charges induce better stability and, therefore, higher transmittance [31,33].

The highest surface charge content was achieved for PFI refining and 50 wt.% oxalic acid treatment, reaching a value of 72.9 µeq/g CMNF. Surface charges represent the anionic nature of the fibers and have been traditionally used to determine the degree of delamination of the pulp fibers for papermaking after disintegration [36].

#### 3.1.3. Morphologies

##### Measurement of Diameter and Length Distributions by Microscopy

The CMNFs obtained with the different treatments were heterogeneous, i.e., they present an ample wide size range. In this work, we analyzed three fractions: (a) diameter greater than 1.0 µm, (b) CMF and (c) CNF.

**Figure 2:**
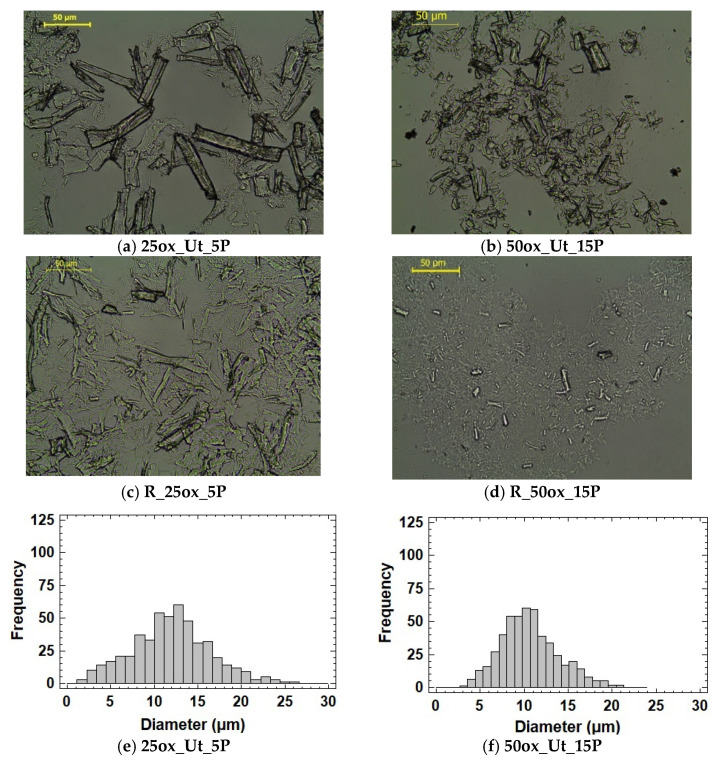

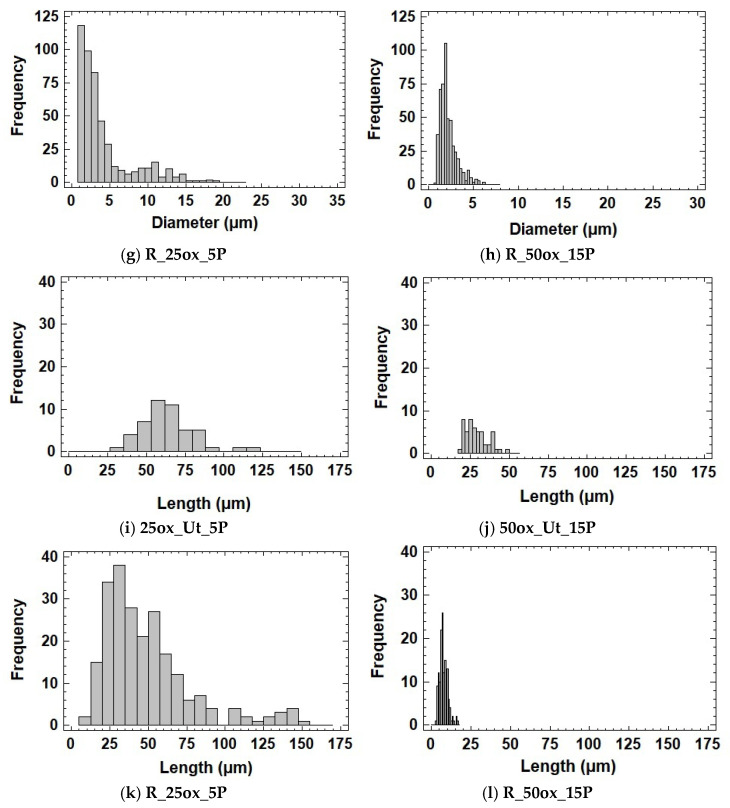
Optical microscopy images of (**a**) 25ox_Ut_5P, (**b**) 50ox_Ut_15P, (**c**) R_25ox_5P and (**d**) R_50ox_15P of CMNF. Distribution of diameter (**e**–**h**) and length (**i**–**l**) of samples fraction of a diameter greater than 1.0 µm.

Table 3 shows the results of average diameter, length and aspect ratio of CMNF fraction of a diameter greater than 1.0 µm determined by optical microscopy (OM). The average diameter values were similar (from 10.7 µm to 11.9 µm) when the rotary homogenizer was used, irrespective of the chemical treatments applied. For pulps refined by PFI mill, the average diameter values were lower (from 2.1 to 3.9 µm). In addition, Table 3 shows that the average length and the aspect ratio decreased with increasing oxalic acid concentration in the chemical treatment. The same effect was observed with the number of passes of the pressurized homogenizer, for both the rotatory homogenizer and refining process. The highest aspect ratio was obtained for R_25ox_5P treatment. Figure 2 shows the OM images and histograms of length and diameter of samples 25ox_Ut_5P, 50ox_Ut_15P, R_25ox_5P and R_50ox_15P. A strong morphological difference is observed between these mechanical treatments. For the rotary homogenization process, cut fibers are observed, whereas for the refining process, slender shavings are observed, indicating a fibrillation effect. The OM images, diameter and length histograms of all CMFs are shown in Figure S1 in the Supplementary Material.

**Figure 3:**
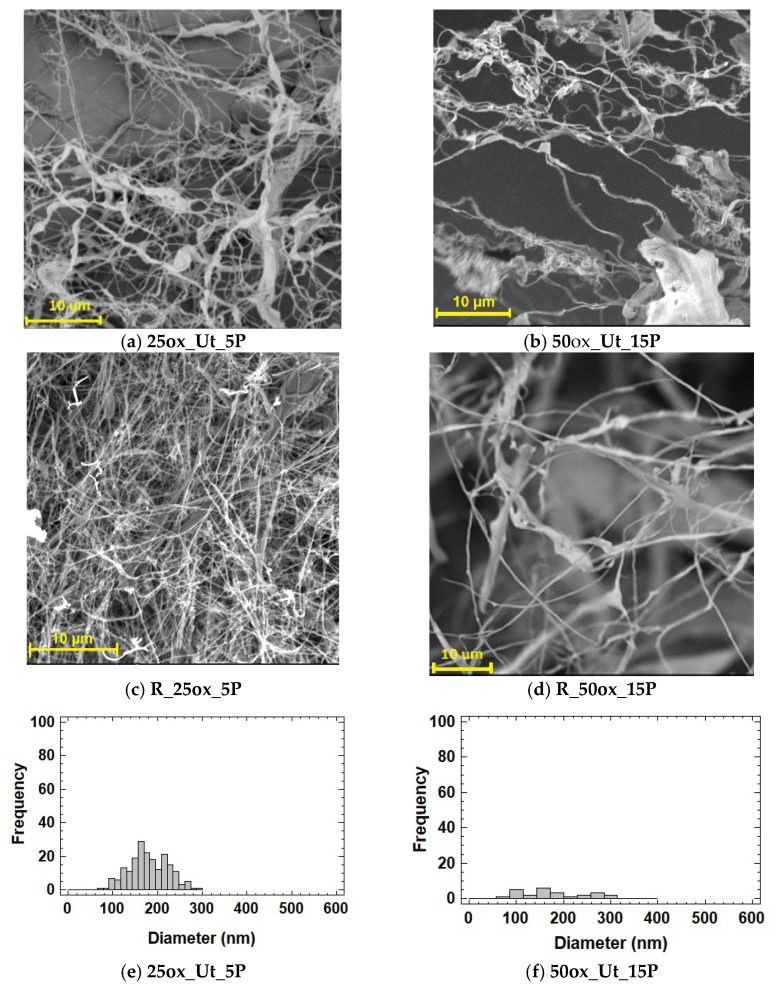

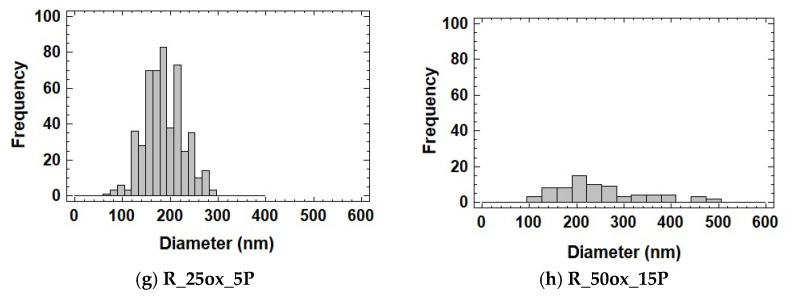
SEM images of (**a**) 25ox_Ut_5P, (**b**) 50ox_Ut_15P, (**c**) R_25ox_5P and (**d**) R_50ox_15P of CMNF. Distribution of diameter (**e**–**h**).

Table 4 shows the results of average diameter of CMFs. Similar values were found for all CMFs (from 179 ± 43 nm to 257 ± 69 nm). Images and diameter histograms of the CMFs determined by SEM are shown in Figure 3. The presence of entangled microfibers was observed; therefore, it was not possible to measure their length, since their beginning and end were undistinguishable. In addition, some lamella and branches were observed, and both elements suggest that the mechanical treatment has not been sufficient to finish separation of the microfibrils. The SEM images and diameter histograms of all CMFs are shown in Figure S2 in the Supplementary Material.

Table 5 shows average diameter, length and aspect ratio of CNF determined by TEM. No significant differences were observed in the average diameters of any CNFs (from 12 ± 3.0 nm to 16 ± 4.8 nm). However, a decrease in the average lengths was observed for 50% oxalic acid treatment (approx. 725 nm) compared to those obtained by 25% oxalic acid treatment (approx. 1250 nm). This result indicates that the chemical pre-treatment with higher concentration of oxalic acid reduced the CNF length, generating a decrease in their aspect ratio.

**Figure 4:**
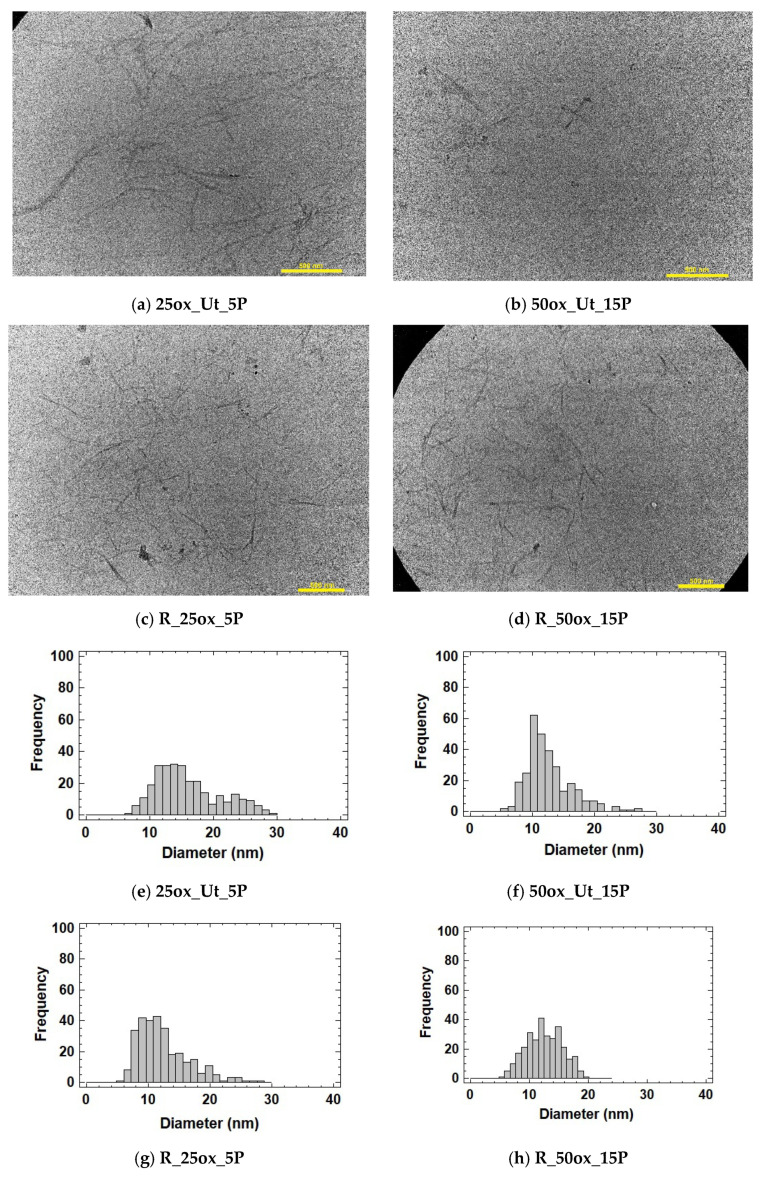

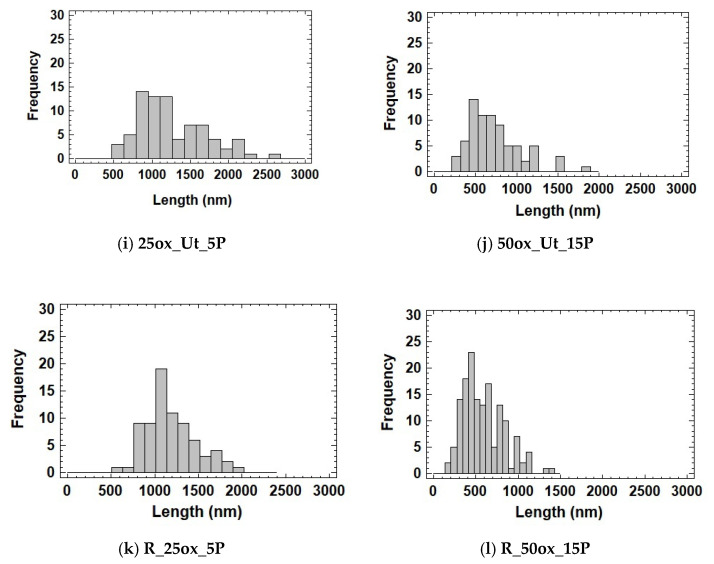
TEM images of (**a**) 25ox_Ut_5P, (**b**) 50ox_Ut_15P, (**c**) R_25ox_5P and (**d**) R_50ox_15P of CMNF. Distribution of diameter (**e–h**) and length (**i–l**).

In addition, pulps treated with PFI showed a higher aspect ratio than those treated with the rotatory homogenizer, and the number of pressurized homogenizer passes did not influence the morphology of the CNFs obtained. Figure 4 shows the TEM images and histograms of CNF samples 25ox_Ut_5P, 50ox_Ut_15P, R_25ox_5P and R_50ox_15P, respectively. The TEM images and diameter histograms of all CNFs are shown in Figure S3 in the Supplementary Material.

##### Sedimentation

**Figure 5:**
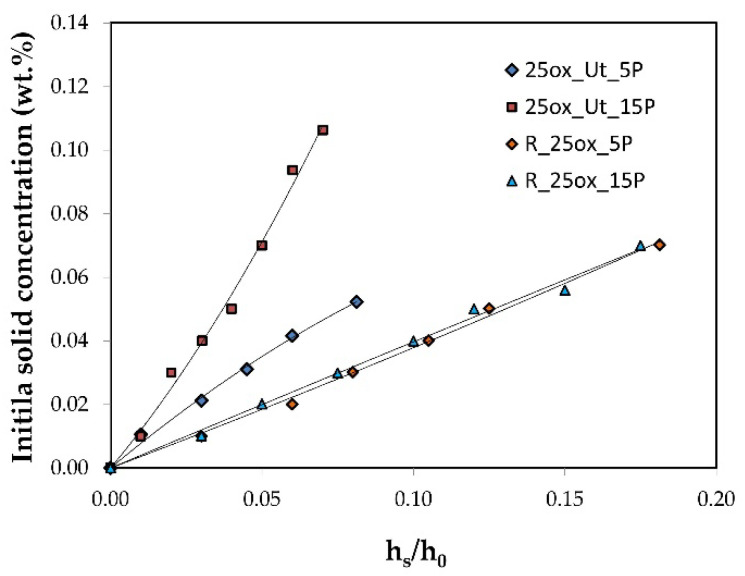
Initial solid concentration vs final sediment height (h_s_)/initial (h_0_) height of suspension.

Figure 5 shows initial concentrations of CMNFs as a function of the ratio of initial (h_o_) to final height (h_s_) of CMNF after 48 h. Data were fitted with a quadratic equation. The first derivative of the curve at the y-intercept gave the gel point (C_c_) and the aspect ratio (A) was estimated using Equation (2) (Table 6). For CMNFs corresponding to 25 wt.% oxalic acid, aspect ratio did not change with increasing number of passes through the homogenizer. In addition, the CNMF obtained by PFI mill shows a higher aspect ratio than the CNMF obtained by rotary homogenizer. The aspect ratio indicated by sedimentation gel point agrees with those measured by TEM (Table 5). It should be noted that, for the sedimentation procedure, both the CMF and CNF fractions were involved, but for TEM, only CNF fractions were considered. This result can be explained by the existence of a similar aspect ratio between the microfibrillar and nanofibrillar fractions. Results indicate that a simple technique such as sedimentation can be used to estimate the aspect ratio of these CNFs. 

However, this technique has some limitations, since the final height of sedimentation cannot be always observed. This occurred for the CMNFs obtained by 50 wt.% oxalic acid treatment, which has a higher content of carboxylic groups and, therefore, higher repulsive forces can prevent their agglomeration and sedimentation.

Sanchez Salvador et al. [36] analyzed the effect of the stability reduction of a CNF suspension and the decrease in a gel point, by changing the pH and the salt concentration of the medium. For this purpose, a CNF of high-charge obtained with a pre-treatment with TEMPO-mediated oxidation was used. They showed that, with the pH value under 4.8 (corresponding to the value of the pKa of COO^−^) and at a high salt concentration (≥5 mM CaCl_2_), the charges decreased and the electrical double layer was compressed, respectively.

##### Shear Viscosity

The aspect ratio was also associated with the intrinsic viscosity of the CMNF suspension. In dilute condition, the interactions between CNMFs were not significant and the rheological properties depended mainly on the morphology of the CNMFs and their entanglements [25,37]. The dilute region is determined by identifying the change in specific viscosity (η_sp_) as a function of the concentration of CNFs in the dispersion [37]. Moreover, the dilute region (Newtonian behavior) and another semi-dilute region above the critical concentration (C*) (power-law region) can be identified [25,38].

**Figure 6:**
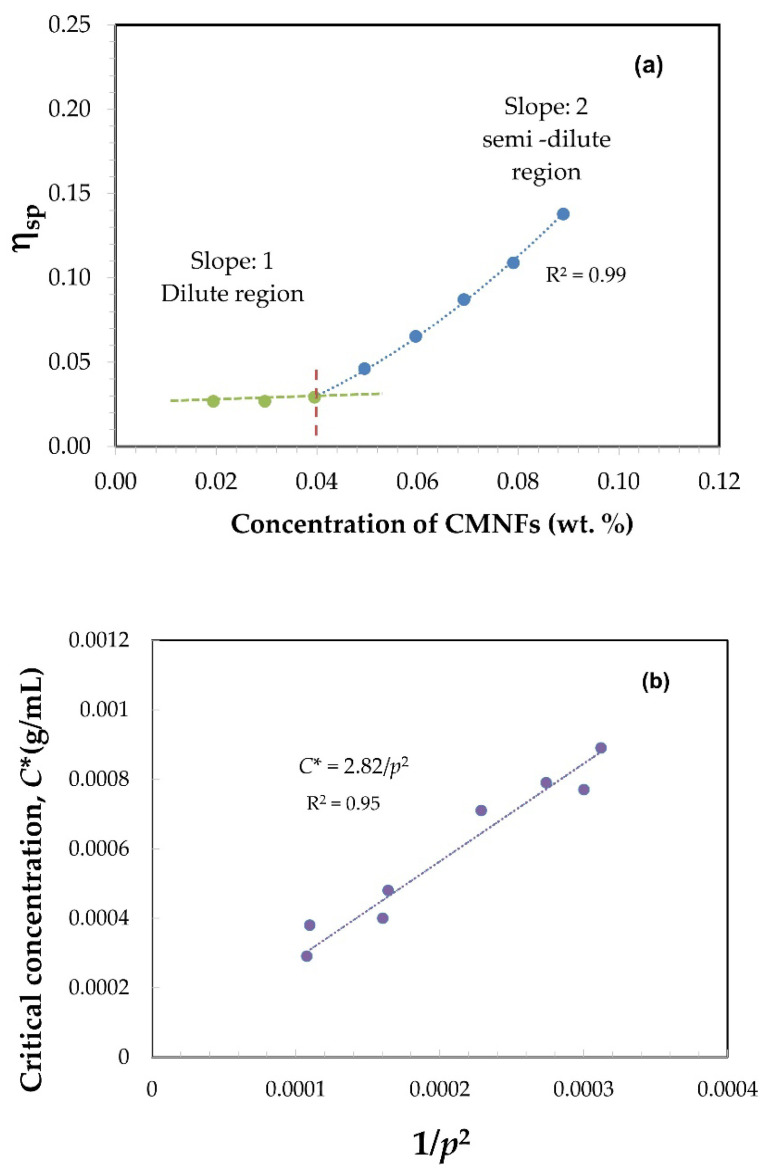
(**a**) Specific viscosity (η_sp_) as a function of the concentration for 25ox_Ut_5P CMNF; (**b**) critical concentration (C*) as a function of the inverse square of the aspect ratio (1/*p*^2^) for all CMNFs analyzed.

Figure 6a shows the plot of specific viscosity (η_sp_) as a function of concentration for 25ox_Ut_5P. It is observed that the C* is clearly defined. The C* values of all CMNFs are shown in Figure S4 in the Supplementary Material. Table 6 shows critical concentration (C*) values of all CMNFs here considered. The CMNFs dispersions obtained by 50 wt.% oxalic acid had a higher critical concentration than 25 wt.% oxalic acid ones. This result can be attributed to the higher quantity of charges incorporated in the chemical pre-treatment. The increase in charge content generates a greater repulsion between the nanofibrils, which makes it possible to increase the dilute region [25]. Furthermore, there was decrease in interfibrillar interactions due to a decrease in the length of the fibrils. 

A relationship between the critical concentration (C*) and the aspect ratio (p) of the CNFs determined by TEM was established (Figure 6b):

$$C^{*}\left[\frac{g}{\mathrm{ml}}\right]=\frac{2.82}{p^{2}}$$

for aspect ratio values between 60–97. The relationship here found is in agreement with that determined by other authors. For instance, Mason [39] found a relationship of C* = 1.5/*p*^2^ for *p*-values between 20–60 for rigid-rod fibers, and Tanaka et al. [29] found C* = 18/*p*^2^ for *p*-values between 90–330 for CNF obtained with (TEMPO)-mediated oxidation. All the relationships show the same trend, but with different slopes. It can be concluded that, as the aspect ratio of the CNFs increases, the slope of the relationship increases. Moreover, Albornoz-Palma et al. [25] analyzed a mechanical CNF with enzymatic pre-treatment and found the relationship C* = 67.0/*p*^2^ for *p*-values between 146–326. The slope of this relationship was greater than that found by Tanaka et al. [29] for CNF obtained by chemical pre-treatment, with similar aspect ratios. They concluded that the type of pre-treatment performed (chemical, enzymatic or mechanic) on the CNF also influences the slope obtained.

**Figure 7:**
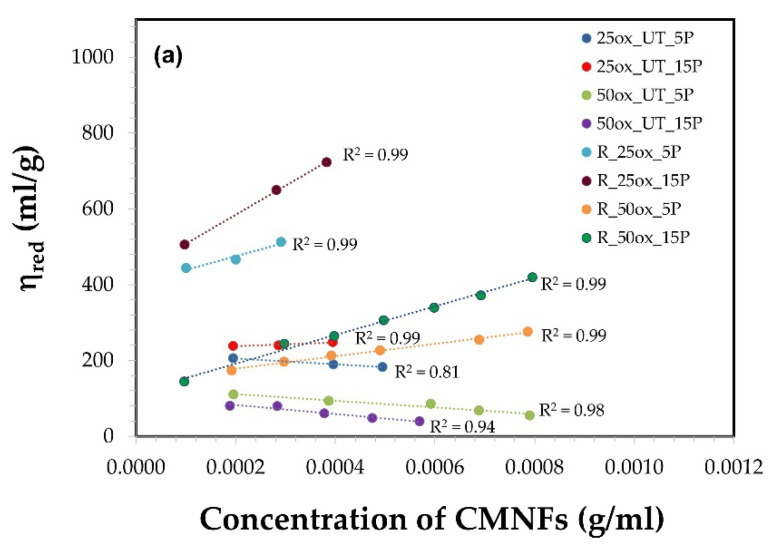

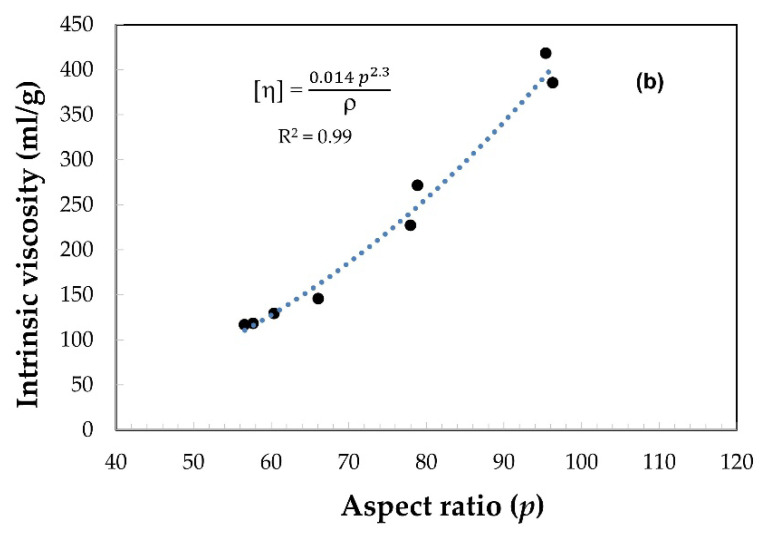
(**a**) Variation in the reduced viscosity according to the concentration of CMNFs in the dilute region, and (**b**) Intrinsic viscosity of the CMNF suspensions as a function of the aspect ratio (p) and the fit with a potential trend represented by a dotted line.

Figure 7a shows the reduced viscosity (η_red_) as a function of concentration of the CMNF dispersions in the dilute region (Newtonian behavior) above the critical concentration. Intrinsic viscosities were obtained by extrapolating the linear fit to zero concentration. The intrinsic viscosity results are shown in Table 7. Higher intrinsic viscosity values were obtained for 25 wt.% oxalic acid CMNFs compared to 50 wt.% oxalic acid ones. No significant difference was detected in the diameters of the CNFs, but their lengths decreased in the nanofibrillar fraction with increasing oxalic acid concentration (Table 5). This length reduction decreased the effective volume of CMNF suspensions, which explains the lower intrinsic viscosity. On the other hand, the CNMFs obtained by 25 wt.% oxalic acid had a lower nanofibrillation yield than 50 wt.% oxalic acid ones. It can be assumed that the nanofibrillar fraction slightly influences the viscosity when the concentration of the suspensions tends to zero. In addition, the CMNFs corresponding to PFI refiner pre-treatment showed a higher intrinsic viscosity than those treated with a rotary homogenizer, for each chemical treatment applied. PFI mill CMNFs had higher surface charge, which can generate an increase in the effective volume.

Figure 7b shows that a strong relationship can be established between intrinsic viscosity and aspect ratio (R^2^ = 0.99):

ρ [η] = 0.014 p^2.3^

where ρ is the density of the CMNFs (1.5 g/mL) that is included to make the intrinsic viscosity dimensionless. This relationship has a higher exponential term than previous records [40]): ρ [η] = 0.15 p^1.9^; and Albornoz-Palma et al. [25]: ρ [η] = 0.051 p^1.85^), indicating greater rigidity of the nanofibers [40]. In addition, a smaller constant of the equation was observed. This constant is related to the nature of the type of CNFs and their configuration in the dispersion [25].

On the other hand, when the aspect ratio of the nanofibers increases, their flexibility increases [41]. Flexible nanofibers subjected to shear stress produce higher hydrodynamic forces (higher viscosity) than rigid nanofiber suspensions [40]. Therefore, it can be concluded that the nanofibers obtained with PFI refining and 25 wt.% oxalic acid have the highest aspect ratio, flexibility and, therefore, intrinsic viscosity.

**Figure 8:**
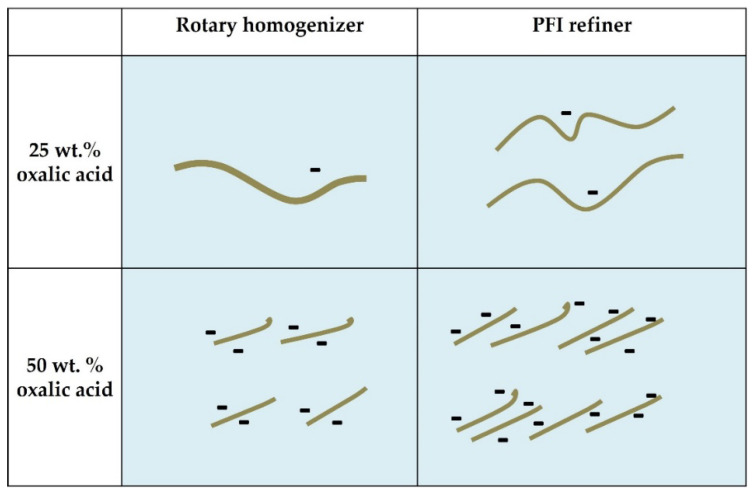
Schematic representation of CNF suspension obtained with different chemical and mechanical pre-treatments.

Figure 8 represents characteristics of the CNFs obtained by different pre-treatments. The number of steps in the pressure homogenizer is not considered because this variable has minor effects. The results of nanofibrillation yield, surface charge, diameter and length were considered in this illustration. The pulp treatment with 50 wt.% oxalic acid leads a higher nanofibrillation yield and surface charges, but shorter length than those treatment with 25 wt.% oxalic acid. This is because the chemical treatment produces two effects: (a) a controlled partial hydrolysis that cuts the cellulose chains and weakens the fibers that break more easily during the fibrillation process in the homogenizer; and (b) a Fischer esterification where carboxylic groups are added to the cellulose. The negative charges of these groups produce repulsive forces between the microfibrils that favors their subsequent separation in the pressure homogenizer. Depending on the application of the CNF, one or another concentration of oxalic acid in the chemical pre-treatment may be more convenient. 

On the other hand, the pulp treated with a PFI mill refiner generates a higher nanofibrillation yield and surface charges and a slight increase in aspect ratio than those pre-treated with a rotary homogenizer. This is because in the pre-treatment with a PFI mill, a greater amount of internal and external fibrillation and less number of cuts are produced, compared to the rotary homogenizer. Therefore, it is more convenient to use the PFI mill for these working parameters.

### 3.2. Film Formation of CMNFs

**Figure 9:**
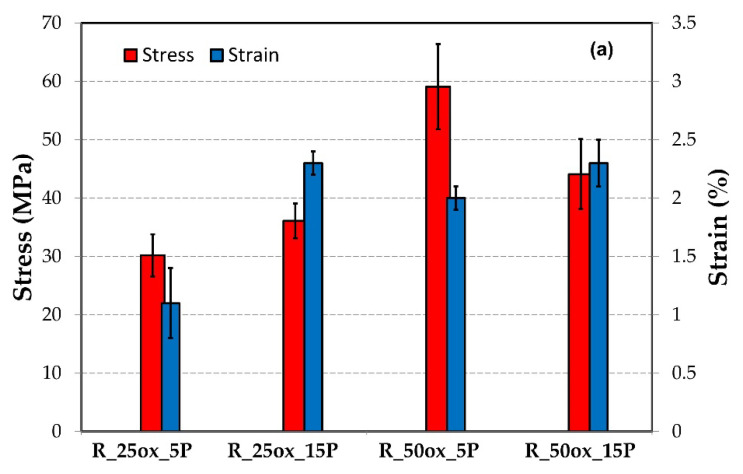

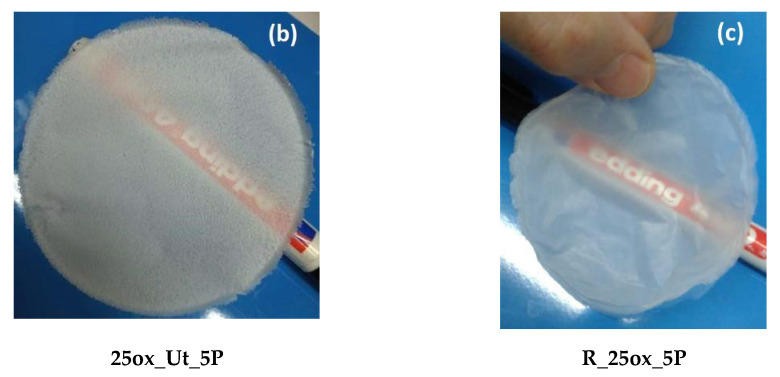
(**a**) Stress and strain of the CNMF films, (**b**,**c**) photographs of 25% oxalic acid CNMF films obtained by pre-treatments with rotary homogenizer and the PFI refiner, respectively.

Figure 9a shows the tensile stress-strain curve of the films formed from CMNFs. The films based on CMNF obtained with the rotary homogenizer were very brittle and it was not possible to test them. Moreover, they were less translucent and homogeneous (Figure 9b, 25ox_Ut_5P) than the films obtained by PFI refiner (Figure 9c, R_25ox_5P). These physical properties can be explained because greater amounts of cut fibers were observed for the CMNFs obtained with a rotary homogenizer, while a greater effect of fibrillation was observed for those obtained by PFI refiner.

Films obtained from 50 wt.% oxalic acid CMNFs had higher tensile strength than films obtained from 25 wt.% oxalic acid. This result indicates that the nanofibrillation yield has more influence on film strength than their aspect ratio.

Neenu et al. (2022) reported mechanical properties of the CNF nanopapers obtained from pineapple pomace pre-treated with oxalic acid. The values obtained (stress: 64.9 MPa and strain: approx. 9%) were similar to those reported here. Moreover, Chen et al. (2020), reported a maximum stress of 49.1 MPa and a strain of 4.5% approximately, for a conductive paper based on TEMPO-CNF and different dosages of silver nanowire (AgNW).

## 4. Conclusions

Based on the results, it was determined that the differences in the pre-treatments strongly influenced the characteristics of the CMNFs obtained. CMNFs obtained employing PFI mill before chemical pre-treatment had better characteristics than those obtained using the rotary homogenizer. On the other hand, 50 wt.% oxalic acid CMNFs showed higher nanofibrillation yield, transmittance, and total and surface charges, but lower aspect ratio than 25 wt.% oxalic acid ones. Nonetheless, the increase in the number of passes through the pressurized homogenizer did not produce significant changes in the characteristics of the CMNFs. Specifically, for CMNFs obtained by 25 wt.% oxalic acid treatment, the aspect ratios calculated by TEM agreed with the gel point values. 

On the other hand, a strong relationship between the intrinsic viscosity of the CMNF dispersions and their aspect ratio was found. Consequently, both parameters, gel point and intrinsic viscosity, can be useful to calculate the aspect ratio of this type of CMNFs. Finally, the tensile strength of CMNF films was more influenced by nanofibrillation yield than their aspect ratio.

## Figures and Tables

**Table 1:** Identifications of cellulose micro/nanofibers according to the treatment made.

Samples	PFI Mill Refining (Revolutions)	Oxalic Acid (wt.%)	Homogenizer Type Ultra-Turrax, 3 min/g Fiber (rpm)	Homogenization at 300 bar (Passes)
**25ox_Ut_5P**	---	25	15,000	5
**25ox_Ut_15P**	---	25	15,000	15
**50ox_Ut_5P**	---	50	15,000	5
**50ox_Ut_15P**	---	50	15,000	15
**R_25ox_5P**	10,000	25	---	5
**R_25ox_15P**	10,000	25	---	15
**R_50ox_5P**	10,000	50	---	5
**R_50ox_15P**	10,000	50	---	15

**Table 2:** Nanofibrillation yield, transmittance and surface charge of CMNFs.

CMNF	Nanofibrillation Yield (%) ^1^	Transmittance (800 nm) (%)	Surface Charge (µeq/g CMNF) ^1^
**25ox_Ut_5P**	4.5 ± 1.2	14.5	10.3 ± 0.9
**25ox_Ut_15P**	5.1 ± 1.6	10.6	11.8 ± 0.1
**50ox_Ut_5P**	19.5 ± 1.4	8.4	18.5 ± 2.1
**50ox_Ut_15P**	20.6 ± 1.6	10.1	30.5 ± 1.5
**R_25ox_5P**	11.6 ± 1.2	18.7	19.6 ± 1.9
**R_25ox_15P**	12.8 ± 1.8	22.4	21.7 ± 0.2
**R_50ox_5P**	54.3 ± 0.33	48.3	72.9 ± 0.3
**R_50ox_15P**	76.5 ± 0.28	72.1	71.0 ± 0.6

^1^ Values are the average of two replicates of the trial.

**Table 3:** Average diameter, length and aspect ratio of original pulp and CMNF fraction of a diameter greater than 1.0 µm determined by optical microscopy.

CMNF	Average Diameter (µm) ^1^	Average Length (µm) ^2^	Average Aspect Ratio (Length/Diameter)
**BEP**	15.6 ± 4.0	945.3 ± 271.4	60.4
**25ox_Ut_5P**	11.9 ± 4.5	63.9 ± 17.2	5.4
**25ox_Ut_15P**	10.6 ± 3.3	42.6 ± 14.9	4.2
**50ox_Ut_5P**	10.9 ± 3.4	32.0 ± 7.8	2.9
**50ox_Ut_15P**	10.7 ± 3.3	29.1 ± 7.2	2.7
**R_25ox_5P**	3.9 ± 3.6	48.7 ± 28.4	14.1
**R_25ox_15P**	3.3 ± 2.5	25.6 ± 13.3	7.8
**R_50ox_5P**	2.8 ± 1.0	11.1 ± 5.5	4.0
**R_50ox_15P**	2.1 ± 1.0	7.7 ± 2.7	3.6

^1^ Values are the average of 500 measurements. ^2^ Values are the average of 50 measurements.

**Table 4:** Average diameter of CMF fraction determined by SEM.

CMNF	Average Diameter (µm) ^1^
**25ox_Ut_5P**	179 ± 43
**25ox_Ut_15P**	257 ± 69
**50ox_Ut_5P**	220 ± 74
**50ox_Ut_15P**	180 ± 71
**R_25ox_5P**	186 ± 40
**R_25ox_15P**	252 ± 68
**R_50ox_5P**	193 ± 95
**R_50ox_15P**	226 ± 69

^1^ Values are the average of 200 measurements.

**Table 5:** Average diameter of CNF determined by TEM.

CNF	Average Diameter CNF (nm) ^1^	Average Length (nm) ^2^	Average Aspect Ratio (Length/Diameter)
**25ox_Ut_5P**	16.0 ± 5.0	1262 ± 443	78.9
**25ox_Ut_15P**	16.0 ± 4.8	1250 ± 430	78.0
**50ox_Ut_5P**	12.0 ± 3.0	725 ± 248	60.4
**50ox_Ut_15P**	12.6 ± 3.7	727 ± 322	57.7
**R_25ox_5P**	12.5 ± 4.2	1204 ± 298	96.3
**R_25ox_15P**	13.2 ± 3.5	1298 ± 569	98.3
**R_50ox_5P**	12.0 ± 3.5	793 ± 227	66.1
**R_50ox_15P**	12.5 ± 3.0	707 ± 183	56.6

^1^ Values are the average of 300 measurements. ^2^ Values are the average of 75 measurements.

**Table 6:** Sedimentation data fitted with a quadratic equation, gel point (C_c_) and aspect ratio (A) of CMNFs according to CN theory.

CMNF	Y = Ax^2^ + Bx ^(^*^)^	Gel Point (C_c_) (wt.%) ^(^**^)^	Aspect Ratio (A) (Length/Diameter)
**25ox_Ut_5P**	y = −1.861x^2^ + 0.794x	0.794	67.3
**25ox_Ut_15P**	y = 10.99x^2^ + 0.793x	0.793	67.4
**R_25ox_5P**	y = 0.178x^2^ + 0.361x	0.361	99.9
**R_25ox_15P**	y = −0.044x^2^ + 0.401x	0.401	94.8

* R^2^ higher than 0.99 for all case, where Y is initial solid concentration and X is (h_s_)/(h_0_). ** The first derivate of the curve at the y-intercept gives the gel point (C_c_).

**Table 7:** The critical concentration (C*) and intrinsic viscosity of CMNFs dispersions.

CMNF	Critical Concentration (C*) CMNF (wt.%)	Intrinsic Viscosity CMNF (mL/g)
**25ox_Ut_5P**	0.040	187.9
**25ox_Ut_15P**	0.048	225.4
**50ox_Ut_5P**	0.079	129.1
**50ox_Ut_15P**	0.077	108.1
**R_25ox_5P**	0.029	367.6
**R_25ox_15P**	0.038	410.5
**R_50ox_5P**	0.071	145.5
**R_50ox_15P**	0.089	115.9

## Data Availability

Not applicable.

## Supplementary Materials

The following supporting information can be downloaded at: https://www.mdpi.com/article/10.3390/nano12172908/s1, Figure S1: OM images (scale of 50 µm) diameter and length distribution of CMF., Figure S2: SEM images (scale of 10 µm), diameter and length distribution of CMF., Figure S3: TEM images (scale of 10 nm), diameter and length distribution of CNF., Figure S4: Variation in the specific viscosity, $\eta_{sp}$ according to the concentration of CNFs.
