# Supplementary material for: Effect of Oxalic Acid Concentration and Different Mechanical Pre-Treatments on the Production of Cellulose Micro/Nanofibers
Source: Nanomaterials (Basel). 2022 Aug 24;12(17):2908. doi: 10.3390/nano12172908 (PMC9457602; doi:10.3390/nano12172908)
Supplement: Supplementary file 1 [file nanomaterials-12-02908-s001.zip › nanomaterials-1876671-supplementary.pdf]

nanomaterials

MDPI

Supplementary material

Effect of Oxalic Acid Concentration and Different Mechanical Pre-Treatments on the Production of Cellulose Micro/Nanofibers

Gabriela Adriana Bastida, Carla Natalí Schnell, Paulina Mocchiutti, Yamil Nahún Solier, María Cristina Inalbon, Miguel Ángel Zanuttini and María Verónica Galván *

Institute of Cellulosic Technology, Faculty of Chemical Engineering (FIQ-CONICET), National University of the Litoral, Santiago del Estero 2654, S3000AOJ, Santa Fe, Argentina

* Correspondence: vgalvan@fiq.unl.edu.ar; Tel./Fax: +54-342-4520019

This file includes **Supplementary Figure S1, S2, S3 and S4**

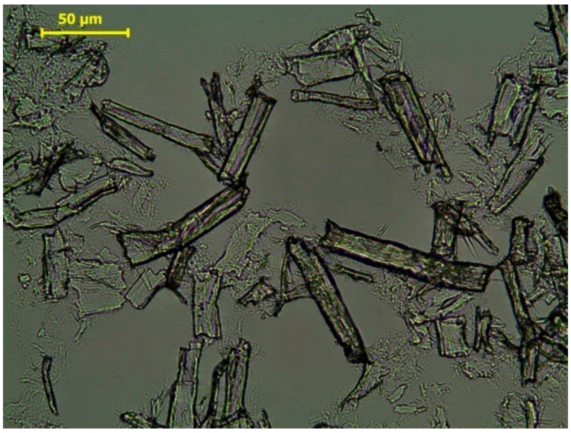

25ox_Ut_5P

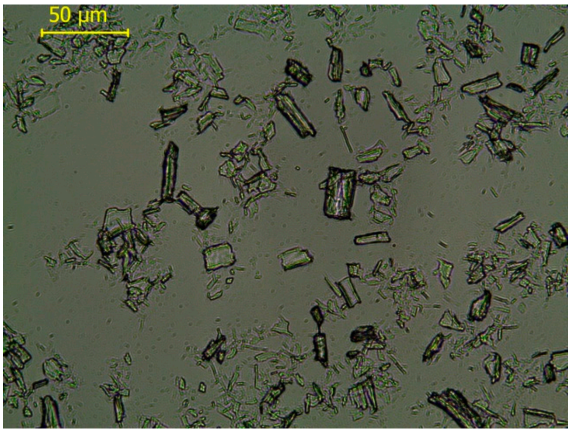

50ox_Ut_5P

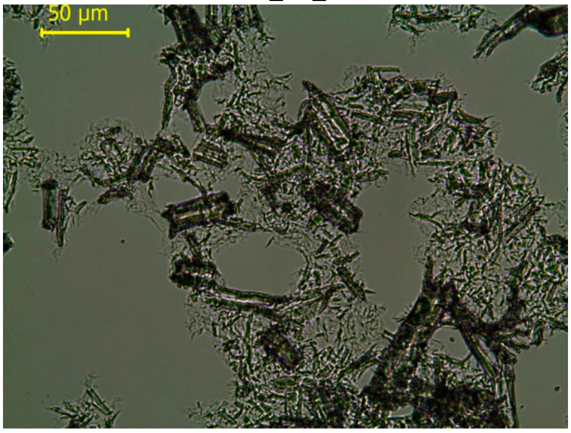

25ox_Ut_15P

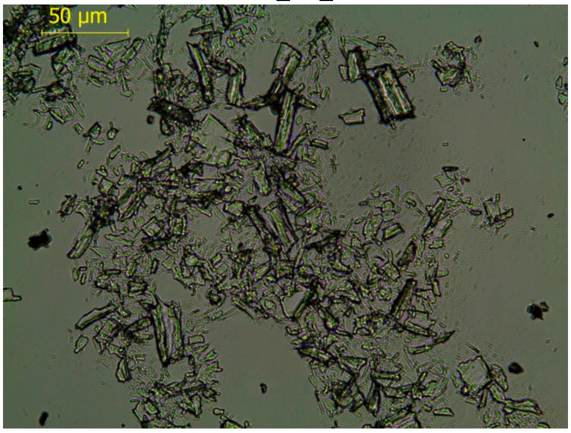

50ox_Ut_15P

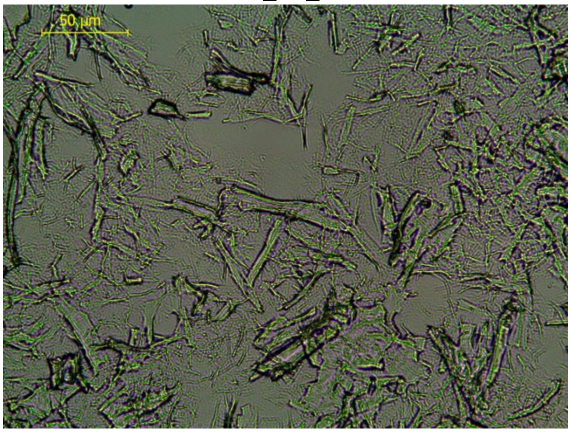

R_25ox_5P

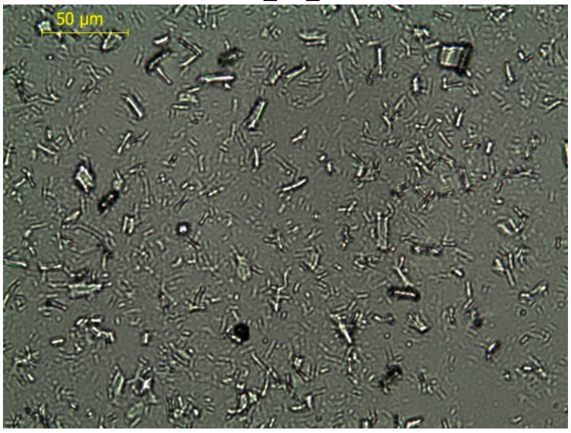

R_50ox_5P

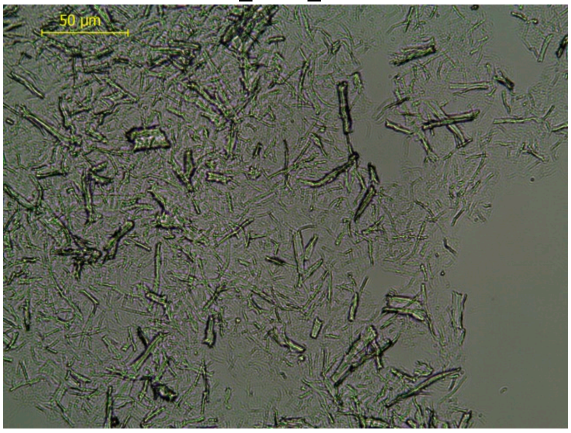

R_25ox_15P

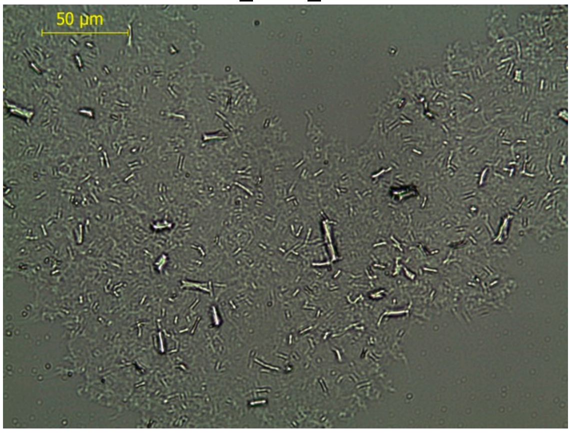

R_50ox_15P

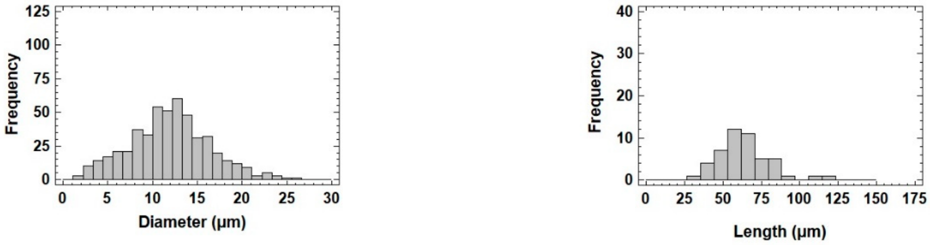

25ox_Ut_5P

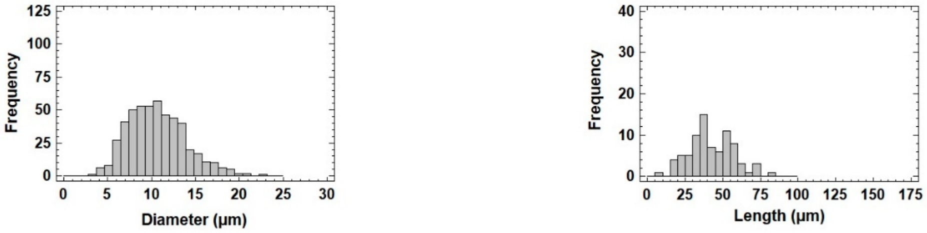

25ox_Ut_15P

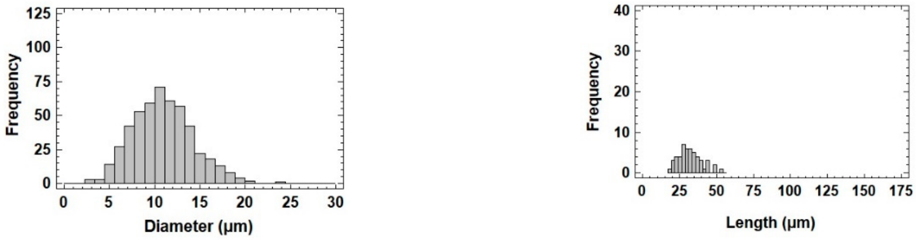

50ox_Ut_5P

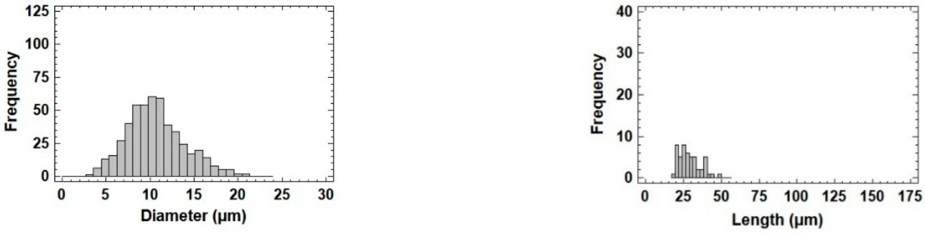

50ox_Ut_15P

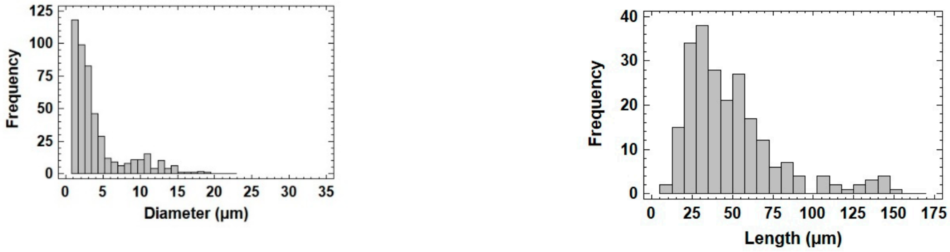

R_25ox_5P

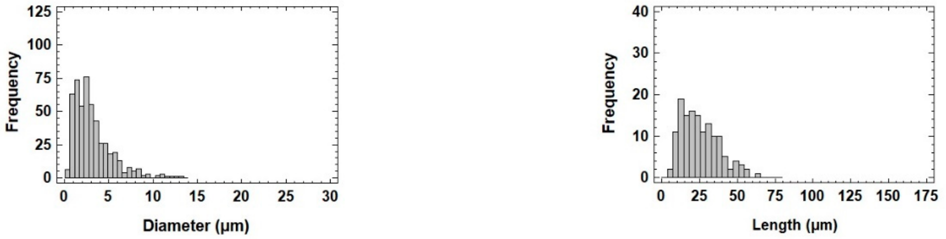

R_25ox_15P

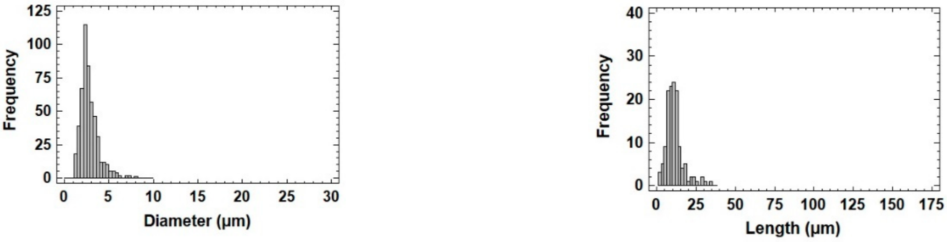

R_50ox_5P

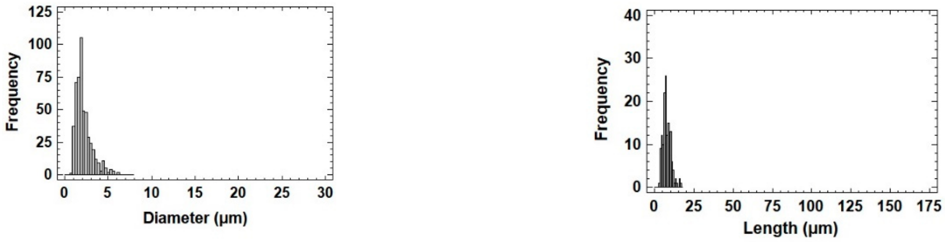

R_50ox_15P

Figure S1: OM images (scale of 50 μm) diameter and length distribution of CMF.

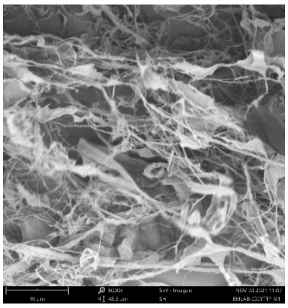

25ox_Ut_5P

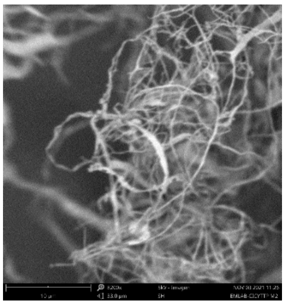

25ox_Ut_15P

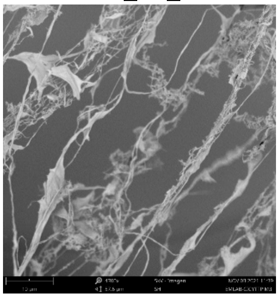

50ox_Ut_5P

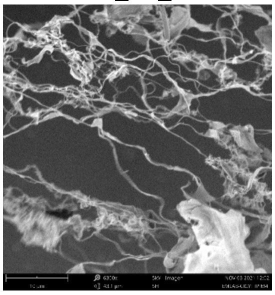

50ox_Ut_15P

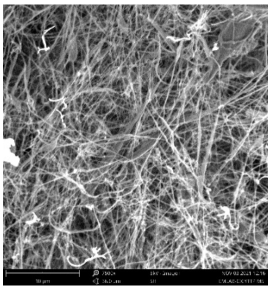

R_25ox_5P

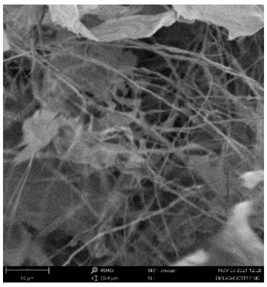

R_25ox_15P

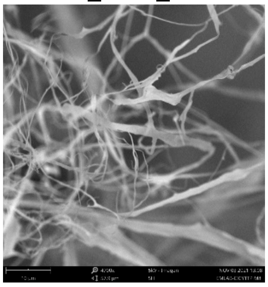

R_50ox_5P

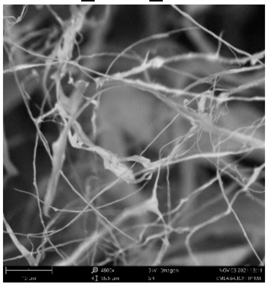

R_50ox_15P

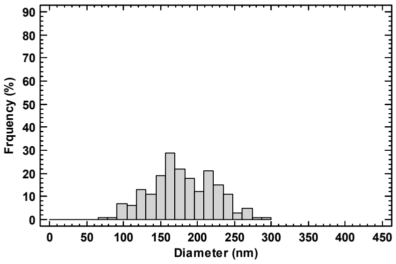

25ox_Ut_5P

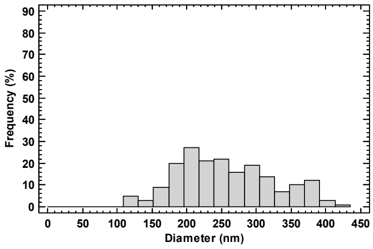

25ox_Ut_15P

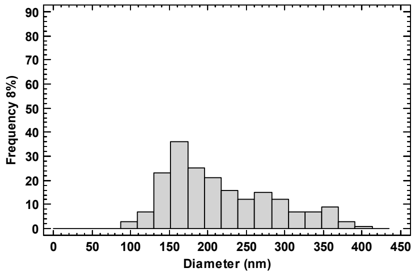

50ox_Ut_5P

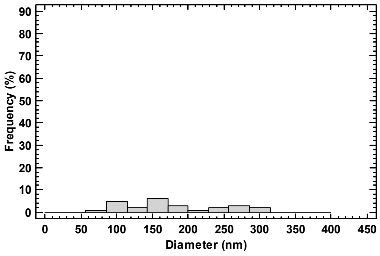

50ox_Ut_15P

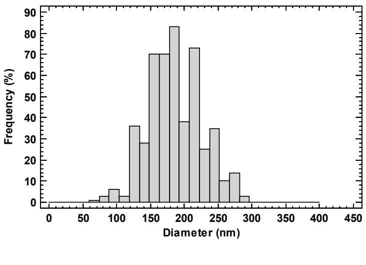

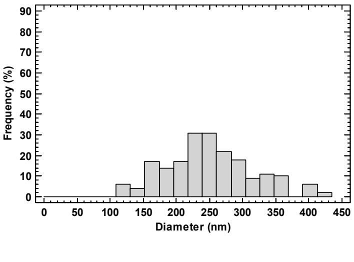

R_25ox_5P

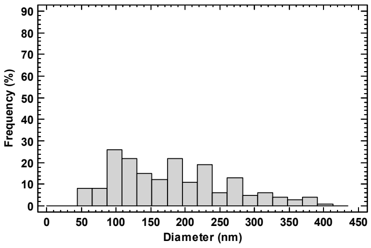

R_25ox_15P

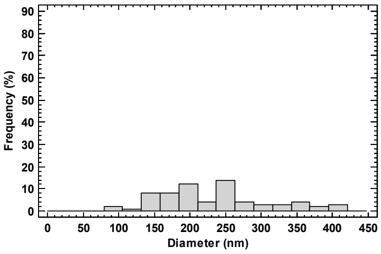

R_50ox_5P

R_50ox_15P

Figure S2: SEM images (scale of 10 μm), diameter and length distribution of CMF.

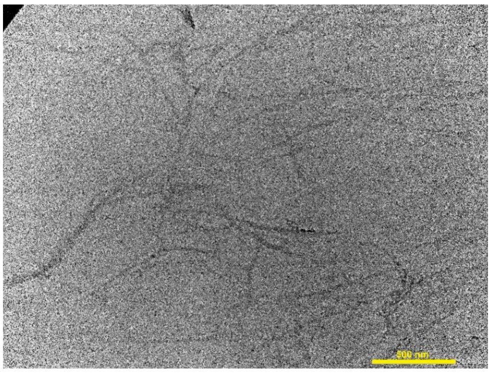

25ox_Ut_5P

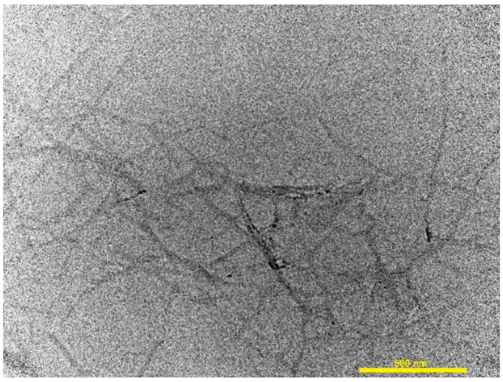

25ox_Ut_15P

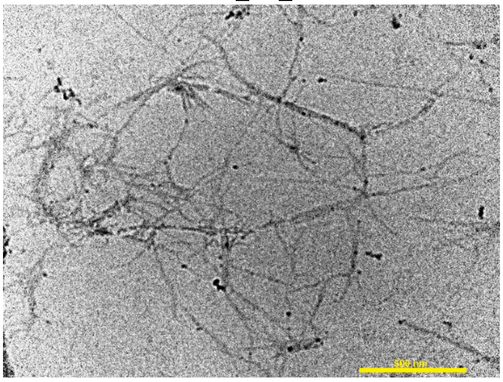

50ox_Ut_5P

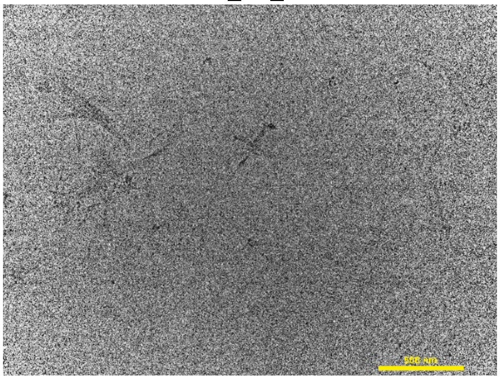

50ox_Ut_15P

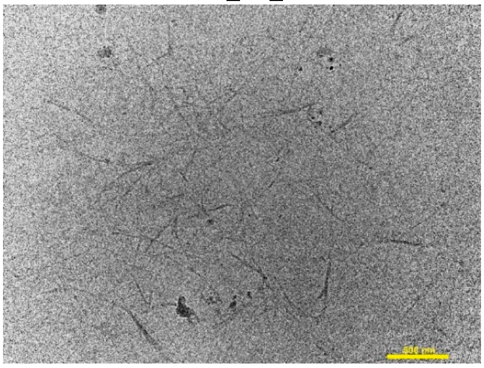

R_25ox_5P

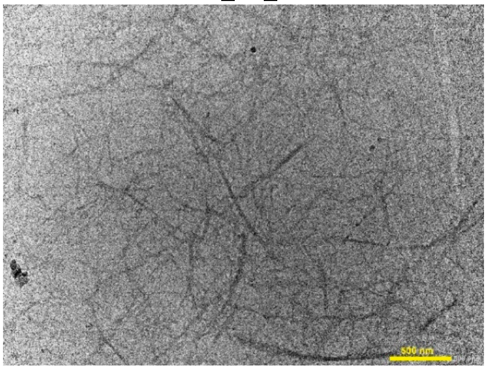

R_25ox_15P

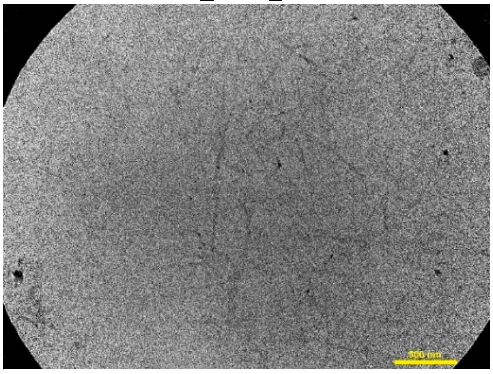

R_50ox_5P

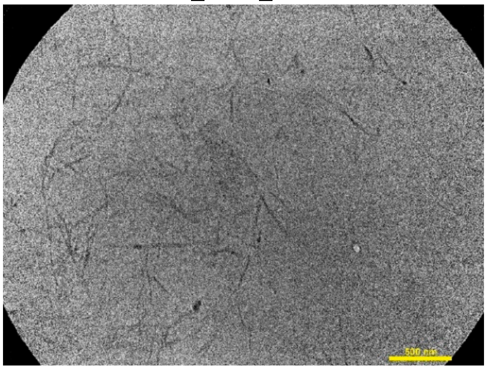

R_50ox_15P

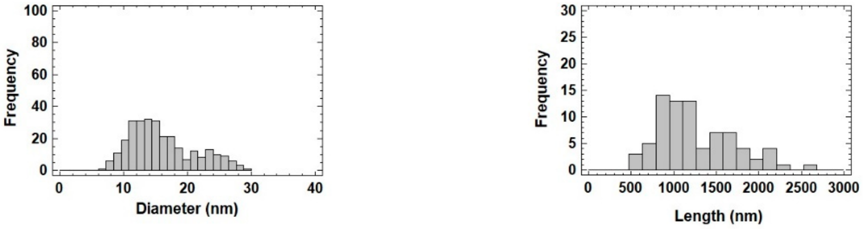

25ox_Ut_5P

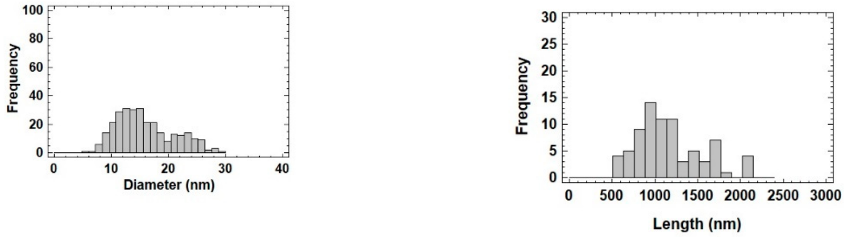

25ox_Ut_15P

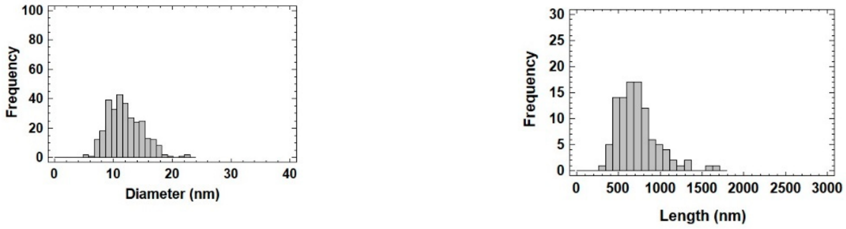

50ox_Ut_5P

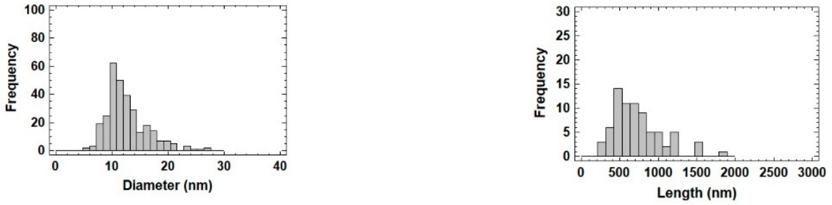

50ox_Ut_15P

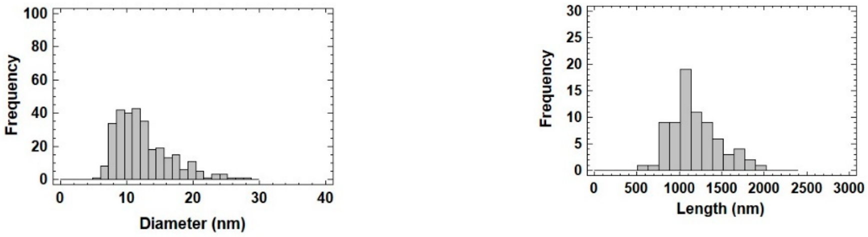

R_25ox_5P

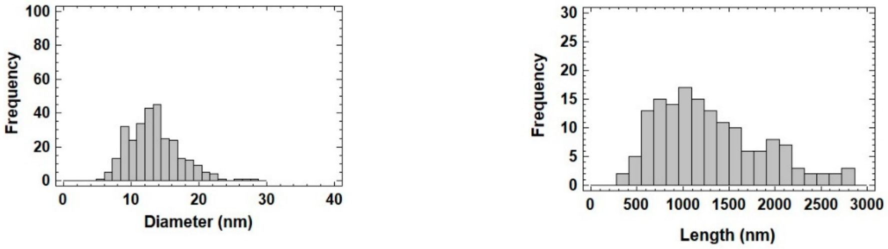

R_25ox_15P

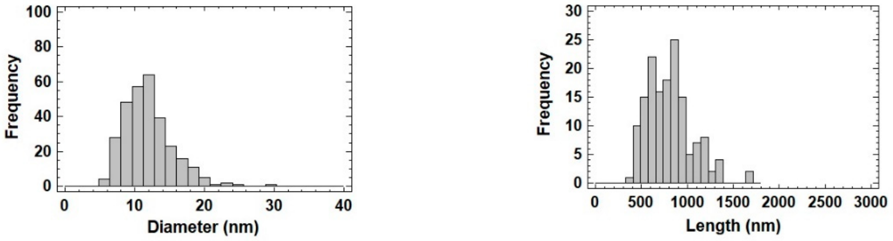

R_50ox_5P

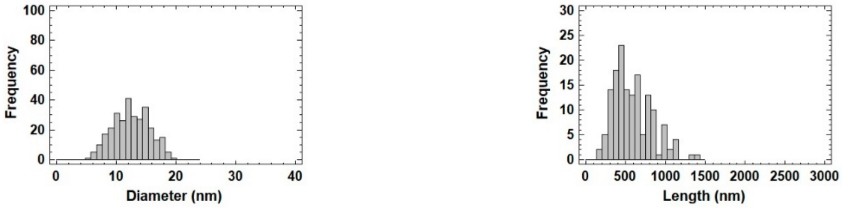

R_50ox_15P

Figure S3: TEM images (scale of 10 nm), diameter and length distribution of CNF.

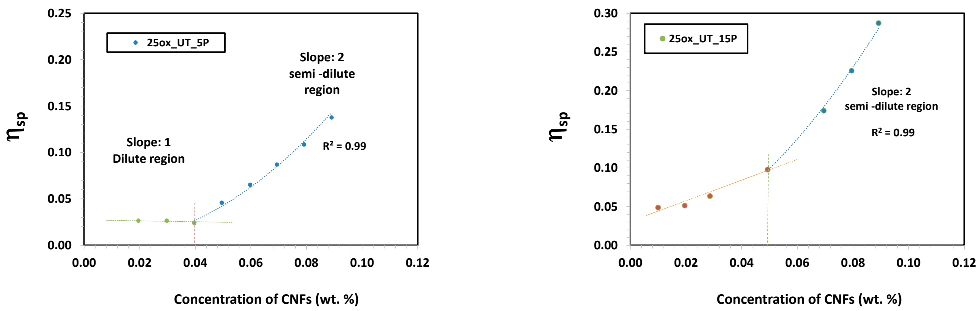

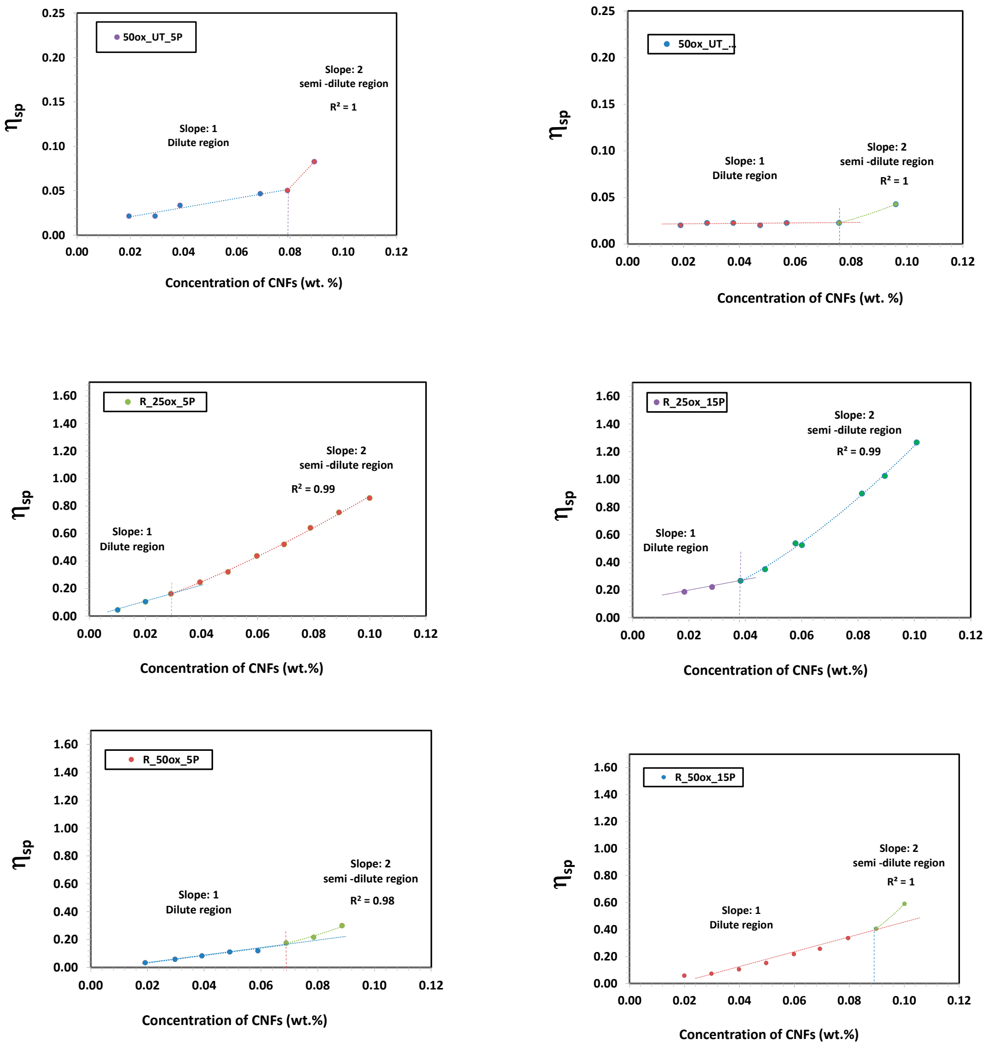

Figure S4: Variation in the specific viscosity, η_{sp} according to the concentration of CNFs.
